# Dynamometry for the measurement of grip, pinch, and trunk muscles strength in subjects with subacute stroke: reliability and different number of trials

**DOI:** 10.1590/bjpt-rbf.2014.0173

**Published:** 2016-07-11

**Authors:** Larissa T. Aguiar, Júlia C. Martins, Eliza M. Lara, Julianna A. Albuquerque, Luci F. Teixeira-Salmela, Christina D. C. M. Faria

**Affiliations:** 1 Departamento de Fisioterapia, Escola de Educação Física, Fisioterapia e Terapia Ocupacional, Universidade Federal de Minas Gerais (UFMG), Belo Horizonte, MG, Brazil

**Keywords:** physical therapy, muscle strength dynamometer, reproducibility of results, stroke

## Abstract

**Background:**

Muscle strength is usually measured in individuals with stroke with Portable dynamometers (gold standard). However, no studies have investigated the reliability, the standard error of measurement (SEM) and the minimal detectable difference (MDD_95%_) of the dynamometry for the measurement of hand grip, pinch grip and trunk strength in subjects with subacute stroke.

**Objective:**

1) To investigate the intra and inter-rater reliability, the SEM and the MDD_95%_ of the portable dynamometers for the measurement of grip, pinch and trunk strength in subjects with subacute stroke, and 2) to verify whether the use of different number of trials (first trial and the average of the first two and three trials) affected the results.

**Method:**

32 subjects with subacute stroke (time since stroke onset: 3.6 months, SD=0.66 months) were evaluated. Hand grip, 3 pinch grips (i.e. pulp-to-pulp/palmar/lateral) and 4 trunk muscles (i.e. flexors, extensors, lateral flexors and rotators) strength were bilaterally assessed (except trunk flexors/extensors) with portable dynamometry by two independent examiners over two sessions (1-2 weeks apart). One-way ANOVAs and intraclass correlation coefficients (ICC_2,k_) were used for analysis (*α*=0.05). SEM and MDD_95%_ were also calculated.

**Results:**

For all muscular groups and sources of outcome values, including one trial, after familiarization, similar results were found (0.01≤F≤0.08; 0.92≤*p*≤0.99) with significant and adequate values of intra-rater (0.64≤ICC≤0.99; 0.23*≤*95%CI*≤*0.99) and inter-rater (0.66≤ICC≤0.99; 0.25*≤*95%CI*≤*0.99) reliability. SEM and MDD_95%_ were considered low (0.39≤EPM≤2.21 Kg; 0.96≤MMD_95%_≤6.12 Kg) for all outcome scores.

**Conclusion:**

Only one trial, following familiarization, demonstrated adequate intra-rater and inter-rater reliability of the portable dynamometers for the measurement of hand grip, pinch grip and trunk strength in subjects with subacute stroke.

## BULLET POINTS

- Portable dynamometers present adequate inter and intra-rater reliability in the subacute phase of stroke.
- One trial provides similar results to those obtained with the average of the first two and three trials.
- In clinical practice, one trial can be used to measure hand and trunk muscle strength.

## Introduction

Hemiparesis, a disability arising from a stroke, with muscle weakness predominant in the hemi-body contralateral to the cerebral lesion, is observed in approximately 77% of individuals with stroke^1^ ^-^ ^4^. Muscle weakness is also observed in the hemi-body ipsilateral to the brain lesion and in the torso as a whole^5^ ^-^ ^8^. Thus, the measurement of muscle strength in post stroke individuals is commonly performed^9^ in different phases of the disease^10^ ^,^ ^11^.

Portable dynamometers are considered a valid instrument for measuring isometric muscular strength - they are easy to use, they provide objective measurements, they are sensitive for detecting changes in muscle strength^10^ ^,^ ^12^, and they provide suitable values of validity and reliability for different muscle groups and populations tested^11^. The intra-rater and inter-rater reliability of portable dynamometers in the measurement of hand grip, pinch grip, and trunk strength was recently investigated in individuals in the chronic phase of stroke^13^, and demonstrated adequate results, with Intraclass Correlation Coefficients (ICC) varying from moderate to very high^14^ (0.58≤ICC≤0.98).

Although commonly regarded as a uniform population, subjects with stroke have unique features in each recovery phase (i.e. acute (first three months), subacute (three to six months), and chronic phases (≥ six months of involvement by stroke))^15^ ^-^ ^17^, which may affect the measurement of muscular strength. Motor recovery and the ability to perform functional activities, such as holding an object or sit-to-stand, are examples of these features^18^ ^,^ ^19^. Therefore, for portable dynamometers to be employed in the subacute phase of stroke, it is necessary to assess measurement properties of these instruments in this population. To our knowledge, only Chen et al.^20^ investigated the reliability of portable dynamometers in individuals in the subacute phase of stroke. However, the authors only assessed intra-rater reliability and only for measurement of handgrip, tripod, and lateral pinch strength. Moreover, the study sample comprised 62 individuals in both subacute and chronic phases of stroke, without separation of the results by phase. According to a recent systematic review, no study has investigated the measurement properties of portable dynamometers to assess hand grip, pinch grip, and trunk strength specifically in the subacute phase of stroke^20^.

Because consistent and reproducible data are essential for credible evaluations, determination of the threshold between error and true change is required for the interpretation of the results. The measurement properties should not be intrinsic characteristics of the measuring instrument^21^. Furthermore, the use of only one trial could facilitate the clinical applicability of the tests. Therefore, the objectives of this study were: (1) to investigate the intra-rater and inter-rater reliabilities of portable dynamometers (SAEHAN® Hydraulic Hand Dynamometer, SAEHAN® Hydraulic Pinch Gauge, and Microfet2® digital manual dynamometer) for the assessment of handgrip, pinch grip (i.e, pulp-to-pulp, tripod, and lateral pinch), and trunk strength; (2) to determine the standard error of the measurement (SEM) and the minimal detectable change (MDC); and (3) to assess whether a different number of trials of measures (i.e. first trial, the average of the first two trials, and the average of three trials) affects the results, in subjects in the subacute phase of stroke.

## Method

### Sample

Individuals from the local community participated in this study. The following inclusion criteria were used: a diagnosis of stroke (subacute phase: between 3 and 6 months since the initial episode)^17^ and age greater than 19 years. Exclusion criteria included those who presented possible cognitive deficits identified by the Mini Mental State Examination based on cutoff points for three levels of education as established by Bertolucci et al.^22^ (i.e. illiterate: 13 points; one to seven years of schooling: 18 points; eight or more years of schooling: 26 points), or the inability to respond to the command “Raise your unaffected arm and open your hand”^23^, or who had pain or another health condition that affected muscular strength of the hands or trunk.

To determine the number of individuals to be assessed, MedCalc® software, version 12.7.5 (MedCalc Software, Ostend, Belgium) was used, with a sample calculation for a correlation coefficient using power=0.8, *r*=0.70, and *α*=0.05. The result was n=14. A prerequisite for use of statistical tests that assess the correlation between variables is the sample variability in relation to the outcome of interest^21^. Taking into account the characteristics that could result in variations in muscle strength, the authors tried to achieve variability in relation to age, sex, and the degree of motor recovery (severe, moderate, or mild) assessed by the Fugl-Meyer Scale for motor function of the Upper Extremity (UE-FM)^24^. Thus, assuming n=14 for each of two age groups that could demonstrate variation in muscular strength (20-64 years and over 64 years), n=28 was calculated. Assuming that approximately 15% of the participants would withdraw on the second day of evaluation, a final sample of 32 (28+4[15%]) participants was determined as an attempting to assure sample variability in muscular strength and improve the statistical robustness. However, it is important to point out that the sample size should be at least 14. Subjects with stroke were included in the present study even though they could not accomplish all muscle groups’ assessments. Therefore, once some subjects were not able to activate some muscle groups, the sample size for each muscle group evaluated varied.

Before data collection, the individuals read and signed an Informed Consent Form approved by the Research Ethics Committee at the Universidade Federal de Minas Gerais (UFMG), Belo Horizonte, MG, Brazil (ETIC 04 92.0.203.000-10). Clinical and demographic data such as age, sex, type and time since stroke, paretic hand, stage of motor recovery (i.e. on Fugl-Meyer scale)^24^, and trunk performance (i.e. Trunk Impairment Scale)^25^, were collected by a physical therapist with prior experience in the assessment of muscular strength of individuals with stroke in the chronic phase and who was trained to meet the objectives of the present study. The paretic upper extremity (UE) was scored using the Modified Ashworth Scale^26^ using flexors of the elbow, wrist, and fingers, and by the decrease in muscle strength, in comparison with the non-paretic UE. However, unlike the UE-FM, the trunk muscles were not identified in this study as paretic and non-paretic, but as right and left sides. This was done because these muscles receive innervation from both brain hemispheres, although predominantly from the contralateral side^27^, and also because the trunk motor deficiencies in individuals with stroke can occur on both sides, with compensation by bilateral innervation^28^.

Bilateral muscles strength of handgrip, pinch grip (i.e. pulp-to-pulp, tripod, and lateral), and trunk muscles (i.e. extensors, flexors and lateral flexors, and rotators) were measured by two trained physical therapists (rater-1 and rater-2) with one year of experience in the assessment of muscular strength using a dynamometer. These raters were trained for this assessment by a physical therapist with four years of training and two years of experience in the assessment of muscular strength of post-stroke individuals using portable dynamometers. Training consisted of a detailed explanation and repeated practice procedures for the assessment of strength of the chosen muscle groups using dynamometers. The evaluation of muscle strength for the collection of data was based on a previous study developed for individuals in the chronic phase of stroke, and included the muscle groups evaluated in the present study, with the same instruments and procedures^13^.

### Instruments

The measurement of muscle strength was performed with three portable dynamometers: SAEHAN® Hydraulic Hand Dynamometer Model HS5001 (SAEHAN Corporation, Korea) was used to measure handgrip strength; SAEHAN® Hydraulic Pinch Gauge Model HS5001 was used to measure the 3 pinch grip strengths (i.e.pulp-to-pulp, tripod, and lateral), and Microfet2® digital manual dynamometer (Hoggan Health Industries, UT, USA), was used to measure trunk strength. All instruments were new and factory-calibrated according to the manufacturer's information manual.

### Procedures

All assessments of muscle strength were performed following the same procedures adopted by Faria et al.^13^, except for the interval between the two evaluation sessions, which in this study was 1 to 2 weeks^17^. The range of 1 to 2 weeks was adopted as being unlikely to show changes in muscle strength during this period in subjects with subacute stroke^17^. Furthermore, with this interval, the rater would probably not remember the values measured on the first day. The data for intra-rater reliability were obtained by a single rater (rater-1) on two evaluation days, and the data for inter-rater reliability were obtained by two raters (rater-1 and rater-2) on the first evaluation day. An interval of five minutes was provided between measurements by the two raters to prevent muscle fatigue, as previously adopted^10^. The reading and recording of measurements of muscle strength were performed by a recorder, who was also trained. Thus, rater-1 and rater-2 had no access to the muscle strength data of the participants. Each rater evaluation lasted in average 20-30 minutes. Therefore, in the first evaluation day the entire muscle strength evaluation lasted around 60 minutes.

The measurements of muscle strength were always obtained in the same order, in an alternating manner, starting with the non-paretic UE and the right side of the trunk. First, a demonstration and familiarization with the equipment and procedures were performed to ensure understanding of the test. Then, three trials at maximum isometric strength were each held for five seconds, and the peak value of the 3 trials was recorded. An interval of 15 to 20 seconds was provided between trials. During the muscle strength test, the rater provided verbal encouragement to the participant to attempt maximum effort.

### Statistical analysis

Descriptive statistics and normality tests (i.e. Shapiro-Wilk) were performed for all outcome measures. One-way analysis of variance *(*ANOVA) was used to compare all forms of operationalization of measures, taking into account the values obtained by rater-1 on day-1. The ICC_2,k_ and 95% confidence interval (CI) were calculated to determine the intra-rater and inter-rater reliabilities, based on the different forms of operationalization of measures: first trial (ICC_2,1_), the average of the first two trials (ICC_2,2_), and the average of three trials (ICC_2,3_). Significant results were classified as follows: very low=0-0.25, low=0.26-0.49, moderate=0.50-0.69, high=0.70-0.89, and very high=0.90-1.00^14^.

The stability of the measurements and the amount of change needed to reflect a real difference were determined by using the SEM and MDC based on 95% CI (MDC_95%_). Both the SEM and MDC_95%_ were calculated according to reliability results and the forms of operationalization of measures^21^. To carry out these analyses, the following formulas were used^16^: SEM = standard deviation × √(1-ICC); MDC_95%_=1.96 × SEM × √2. The statistical package SPSS version 15.0 (SPSS Inc., Chicago, IL, USA) (*α*=0.05) was used for the analysis.

## Results

Thirty-two individuals in the subacute phase of stroke (18 men & 14 women), with a mean age of 63±12 years and a mean time following stroke of 3.6±0.66 months were assessed (Table 1). Of these 32, only 24 agreed to participate on the second day of evaluation for work or health reasons.

**Table 1:** Participant’s characteristics of 32 subacute stroke subjects.

**Characteristics**	***n*=32**
Age (years): mean (SD); range [min-max]	63 (12); [35-85]
Time since the onset of stroke (months): mean; range [min-max]	3.6 (0.66); [3-5]
Body mass index (Kg/m^2^): mean (SD); range [min-max]	25 (4.3); [15-37]
Gender	
Men: number (percentage)	18 (56%)
Paretic Side	
Right: number (percentage)	17 (53%)
Type of stroke	
Ischaemic: number (percentage)	30 (94%)
Haemorrhagic: number (percentage)	1 (3%)
Ischaemic and Haemorrhagic: number (percentage)	1 (3%)
Upper Limb Motor Impairment (Fugl-Meyer Scale). score (0-66)	
Mild motor impairments: number (percentage)	24 (75%)
Moderate: number (percentage)	1 (3%)
Severe: number (percentage)	7 (22%)
Trunk Performance scale: median (IQR). score (0-23)	16 (6)

SD: Standard Deviation; IQR: Interquartil Range.

Table 2 presents the results of descriptive statistics, as well as of the ANOVA for comparison of all forms of operationalization of measures for all muscle groups assessed. The values found for all forms of operationalization of measures were similar (0.01*≤*F*≤*0.08; 0.92*≤*p*≤*0.99).

**Table 2:** Results of descriptive statistics (means±SD) and ANOVA regarding the comparisons between the various sources of outcome scores for muscular strength measurement of both the non-paretic and paretic upper extremities and trunk with portable dynamometry (Kg) for 32 subacute stroke subjects.

**Muscular groups (*n*)**	**First trial**	**Mean of two first trials**	**Mean of three trials**		**ANOVA (*F*; *p*)**
**Non-paretic upper extremity**					
Hand grip (25)	28.12±10.48	27.76±10.45	27.41±10.29		0.03; 0.98
Pulp-to-pulp pinch (23)	4.25±1.63	4.22±1.55	4.20±1.51		0.02; 0.98
Tripod pinch (23)	5.63±2.31	5.58±2.03	5.56±1.92		0.01; 0.99
Lateral pinch (23)	7.09±2.10	7.27±2.01	7.22±1.97		0.02; 0.98
**Paretic upper extremity**					
Hand grip (18)	26.31±9.86	25.62±9.97	25.54±10.06		0.01; 0.99
Pulp-to-pulp pinch (17)	4.08±1.44	3.88±1.54	3.89±1.56		0.01; 0.99
Tripod pinch (17)	4.88±2.29	5.02±2.22	4.94±2.09		0.04; 0.97
Lateral pinch (19)	6.32±2.84	6.16±2.77	6.11±2.72		0.01; 0.99
**Trunk**					
Anterior trunk flexors (20)	9.39±3.48	9.28±3.56	9.25±3.48	0.01; 0.99	
Trunk extensors (20)	10.26±3.23	10.16±3.12	9.87±3.25	0.08; 0.92	
Right lateral trunk flexors (20)	8.32±2.85	8.35±2.96	8.51±2.99	0.03; 0.98	
Left lateral trunk flexors (20)	8.43±2.76	8.64±2.73	8.62±2.74	0.04; 0.97	
Right trunk rotators (20)	7.71±2.58	7.46±2.51	7.44±2.56	0.07; 0.93	
Left trunk flexors (20)	7.46±2.69	7.34±2.65	7.22±2.58	0.04; 0.96	

SD: Standard deviation.

Tables 3 and 4 present the values of the ICC with the 95% CI for the intra-rater and inter-rater reliabilities for all muscle groups assessed and forms of operationalization of measures. All measures of intra-rater and inter-rater reliability displayed significant values, classified as moderate to very high (0.64*≤*ICC*≤*0.99; p*≤*0.001) for all muscle groups (Table 3 and 4).

**Table 3:** Intra-class correlation coefficients (ICC) and 95% confidence interval (CI) for the intra-rater reliability for muscular strength measurement of both the non-paretic and paretic upper extremities and trunk with portable dynamometers (Kg) considering various sources of outcome scores for 24 subacute stroke patients.

**Muscular groups** **(*n*)**	**Intra-rater reliability**		
	**First trial**	**Mean of two first trials**	**Mean of three trials**
	**ICC_2.1_ [95% CI]**	**ICC_2.2_ [95% CI]**	**ICC_2.3_ [95% CI]**
**Non-paretic upper extremity**			
Hand grip (17)	0.95^*^ [0.87-0.98]	0.98^*^ [0.94-0.99]	0.99^*^ [0.96-0.99]
Pulp-to-pulp pinch (16)	0.64^**^ [0.23-0.86]	0.83^*^ [0.50-0.94]	0.85^*^ [0.57-0.95]
Tripod pinch (16)	0.84^*^ [0.60-0.94]	0.93^*^ [0.80-0.98]	0.95^*^ [0.85-0.98]
Lateral pinch (16)	0.84^*^ [0.60-0.94]	0.93^*^ [0.79-0.98]	0.94^*^ [0.84-0.98]
**Paretic upper extremity**			
Hand grip (13)	0.97^*^ [0.90-0.99]	0.99^*^ [0.96-0.99]	0.98^*^ [0.95-0.99]
Pulp-to-pulp pinch (12)	0.82^*^ [0.51-0.95]	0.89^*^ [0.63-0.97]	0.90^*^ [0.67-0.97]
Tripod pinch (12)	0.85^*^ [0.57-0.91]	0.95^*^ [0.81-0.98]	0.95^*^ [0.81-0.98]
Lateral pinch (14)	0.91^*^ [0.74-0.97]	0.95^*^ [0.85-0.99]	0.96^*^ [0.88-0.99]
**Trunk**			
Anterior trunk flexors (20)	0.87^*^ [0.71-0.95]	0.94^*^ [0.85-0.98]	0.94^*^ [0.85-0.98]
Trunk extensors (20)	0.79^*^ [0.55-0.91]	0.89^*^ [0.70-0.96]	0.90^*^ [0.71-0.96]
Right lateral trunk flexors (20)	0.84^*^ [0.51-0.94]	0.94^*^ [0.84-0.98]	0.96^*^ [0.89-0.98]
Left lateral trunk flexors (20)	0.88^*^ [0.72-0.95]	0.96^*^ [0.90-0.98]	0.96^*^ [0.90-0.98]
Right trunk rotators (20)	0.77^*^ [0.39-0.91]	0.89^*^ [0.69-0.96]	0.89^*^ [0.72-0.96]
Left trunk rotators (20)	0.74^*^ [0.46-0.89]	0.92^*^ [0.79-0.97]	0.94^*^ [0.84-0.98]

* *p*≤0.001.

** *p*=0.003.

**Table 4:** Intra-class correlation coefficients (ICC) and 95% confidence interval (CI) for the inter-rater reliability for muscular strength measurement of both the non-paretic and paretic upper extremities and trunk with portable dynamometers (Kg) considering various sources of outcome scores for 32 subacute stroke patients.

**Muscular groups** **(*n*)**	**Inter-rater reliability**		
	**First trial**	**Mean of two first trials**	**Mean of three trials**
	**ICC_2.1_ [95% CI]**	**ICC_2.2_ [95% CI]**	**ICC_2.3_ [95% CI]**
**Non-paretic upper extremity**			
Hand grip (24)	0.95^*^ [0.89-0.98]	0.98^*^ [0.95-0.99]	0.98^*^ [0.96-0.99]
Pulp-to-pulp pinch (22)	0.70^*^ [0.41-0.87]	0.84^*^ [0.61-0.93]	0.85^*^ [0.64-0.94]
Tripod pinch (22)	0.86^*^ [0.69-0.94]	0.94^*^ [0.85-0.97]	0.95^*^ [0.88-0.98]
Lateral pinch (22)	0.91^*^ [0.80-0.96]	0.93^*^ [0.84-0.97]	0.95^*^ [0.88-0.98]
**Paretic upper extremity**			
Hand grip (18)	0.95^*^ [0.88-0.98]	0.98^*^ [0.95-0.99]	0.99^*^ [0.96-0.99]
Pulp-to-pulp pinch (17)	0.70^*^ [0.34-0.88]	0.92^*^ [0.78-0.97]	0.94^*^ [0.83-0.98]
Tripod pinch (17)	0.88^*^ [0.69-0.95]	0.94^*^ [0.84-0.98]	0.94^*^ [0.84-0.98]
Lateral pinch (18)	0.88^*^ [0.72-0.96]	0.93^*^ [0.80-0.97]	0.95^*^ [0.85-0.98]
**Trunk**			
Anterior trunk flexors (26)	0.76^*^ [0.37-0.90]	0.90^*^ [0.59-0.96]	0.91^*^ [0.52-0.97]
Trunk extensors (26)	0.66^*^ [0.14-0.86]	0.84^*^ [0.25-0.95]	0.84^*^ [0.21-0.95]
Right lateral trunk flexors (25)	0.87^*^ [0.74-0.94]	0.92^*^ [0.79-0.97]	0.87^*^ [0.64-0.95]
Left lateral trunk flexors (25)	0.70^*^ [0.33-0.87]	0.85^*^ [0.61-0.94]	0.85^*^ [0.59-0.94]
Right trunk rotators (25)	0.73^*^ [0.22-0.90]	0.83^*^ [0.41-0.94]	0.85^*^ [0.49-0.94]
Left trunk rotators (25)	0.78^*^ [0.52-0.90]	0.87^*^ [0.70-0.95]	0.89^*^ [0.76-0.95]

* *p*≤0.001.

Table 5 presents the results of the SEM and MDC_95%_, based on the intra-rater and inter-rater reliabilities and forms of operationalization of the measures. The values of the SEM and MDC_95%_ were similar between the different forms of operationalization of the measures. All SEM (0.39*≤*SEM *≤*2.21 Kg) and MDC_95%_ (0.96*≤*MMD_95%_ *≤*6.12 Kg) values are presented in Table 5.

**Table 5:** Standard errors of measurement (SEM) (Kg) and the minimal detectable difference (Kg) based on 95% confidence interval (MMD_95%_) considering the results of intra-rater and inter-rater reliabilities and the various sources of outcome scores for 24 (intra-rater reliability) and 32 (inter-rater reliability) subacute stroke patients.

	**Intra-rater reliability (n=24)**							**Inter-rater reliability (n=32)**					
**Muscular groups**	**First trial**		**Mean of first two trials**		**Mean of three trials**			**First trial**		**Mean of first two trials**		**Mean of three trials**	
	**SEM**	**MMD_95%_**	**SEM**	**MMD_95%_**	**SEM**	**MMD_95%_**		**SEM**	**MMD_95%_**	**SEM**	**MMD_95%_**	**SEM**	**MMD_95%_**
**Non-paretic upper extremity**													
Hand grip	2.21	6.12	1.38	3.84	0.99		2.74	2.07	5.74	1.27	3.51	1.29	3.57
Pulp-to-pulp pinch	0.79	2.20	0.54	1.51	0.50		1.38	0.80	2.21	0.58	1.62	0.56	1.56
Tripod pinch	0.84	2.33	0.50	1.39	0.41		1.15	0.73	2.01	0.45	1.23	0.41	1.12
Lateral pinch	0.82	2.27	0.54	1.48	0.50		1.38	0.60	1.68	0.52	1.44	0.44	1.22
**Paretic upper extremity**													
Hand grip	1.68	4.64	0.95	2.63	1.33		3.69	2.18	6.03	1.33	3.69	0.93	2.58
Pulp-to-pulp pinch	0.62	1.73	0.52	1.43	0.49		1.36	0.73	2.01	0.39	1.07	0.35	0.96
Tripod pinch	0.87	2.39	0.51	1.41	0.50		1.39	0.79	2.19	0.53	1.47	0.54	1.49
Lateral pinch	0.81	2.26	0.60	1.66	0.52		1.45	0.70	1.95	0.55	1.53	0.46	1.26
**Trunk**													
Anterior trunk flexors	1.23	3.42	0.84	2.33	0.83		2.31	1.78	4.94	1.18	3.28	1.13	3.13
Trunk extensors	1.56	4.31	1.12	3.10	1.06		2.94	2.17	6.00	1.47	4.09	1.45	4.02
Right lateral trunk flexors	1.16	3.20	0.71	1.98	0.59		1.62	1.05	2.91	0.84	2.34	1.07	2.97
Left lateral trunk flexors	0.96	2.66	0.55	1.51	0.54		1.51	1.67	4.63	1.15	3.20	1.19	3.29
Right trunk rotators	1.15	3.19	0.77	2.15	0.80		2.21	1.29	3.57	1.04	2.89	0.99	2.73
Left trunk rotators	1.30	3.61	0.69	1.90	0.59		1.63	1.41	3.91	1.06	2.93	0.96	2.65

## Discussion

The portable dynamometers presented adequate intra-rater and inter-rater reliabilities for measurement of grip, pinch, and trunk muscular strength in individuals in the subacute phase of stroke, with low values of SEM and MDC. In general, the 95% CI of the ICC for intra-rater and inter-rater reliabilities displayed adequate values. Moreover, results obtained with one trial were similar to those obtained with the average of the first two and three trials.

As the ICC is the most appropriate method for analyzing reliability, only studies that used the ICC were used in this discussion. Faria et al.^13^ investigated the intra-rater and inter-rater reliabilities of portable dynamometers in assessing the strength of the same muscle groups evaluated in this study, but with individuals in the chronic phase of stroke. The results of the reliabilities in the chronic phase of stroke were classified as moderate to very high (0.58 *≤* ICC *≤* 0.98)^13^. These results are similar to those of the present study: (0.64 *≤* ICC *≤* 0.99). It was not possible to compare the 95%CI of the ICC with the study of Faria et al.^13^ because that analysis was not reported. Chen et al.^20^, with a sample of individuals in the subacute and chronic phases grouped together, reported an ICC classified as very high (0.96 *≤* ICC *≤* 0.98; 0.93 *≤* 95% CI *≤* 0.99) when evaluating handgrip strength, tripod pinch, and lateral pinch. The result of the present study was similar to that of Chen et al.^20^ for the same muscle groups and forms of operationalization (average of three trials) (0.94 *≤* ICC *≤* 0.99; 0.81 *≤* 95% CI *≤* 0.99). With regard to the use of different forms of operationalization of the measures, the results of the present study were also similar to several published studies with individuals in the chronic phase of stroke^13^ ^,^ ^29^ ^-^ ^32^. In these studies, only one trial, after familiarization, presented similar results for the measurement properties investigated and for the values obtained when compared to other forms of operationalization, except for the measurement of the plantar flexor muscle strength of the non-paretic side using the Modified Sphygmomanometer Test (MST) measured by two raters. Nevertheless, the present study is the first to consider a sample of individuals in the subacute phase of stroke.

All SEM values in this study were below 2.21 Kg, indicating that the measurement of muscular strength by portable dynamometers was able to distinguish between small real changes in muscle strength and error in individuals in the subacute phase of stroke. In addition, the results of the SEM were similar to those reported by Bertrand et al.^33^ and Boissy et al.^34^, who determined the SEM of handgrip muscular strength with a dynamometer, allowing for ICC values of intra-rater reliability, in individuals in the chronic phase of stroke. The MDC indicates the least amount of change that can be interpreted as either real improvement or worsening^21^. The MDC values in this study varied from 0.96 to 6.12 Kg. Therefore, for a change in muscle strength to be considered real after an intervention, when evaluated using portable dynamometers, one must have changes equal to or greater than 0.96 to 6.12 Kg (depending on the muscle group and the number of trials performed). No study was found that investigated the MDC to evaluate muscular strength with a dynamometer in subjects with stroke. However, the values of MDC found were similar to those of healthy individuals (MDC=5.7 and 7.1 Kg), and were lower than the values of individuals hospitalized in intensive care units (MDC=7.8 and 12.5 Kg), whose handgrip strength was evaluated using a dynamometer (average of three trials), taking into account test-test reliability^35^ ^,^ ^36^. The MDC values should be used in interpretation of the measurements of muscle strength using dynamometers in subjects with stroke.

One of the limitations of this study was the fact that the sample comprised few individuals with moderate/severe motor involvement, and had more individuals with the mean time following the stroke closer to three than six months. In addition, three measures of intra-rater reliability on the paretic side (handgrip, pulp-to-pulp, and tripod pinch) had a sample size smaller than 14, the minimum value estimated by the sample size calculation, as it was not possible to reassess the ability of some study participants to exert force with these muscle groups. However, the values of intra-rater reliability for these measures were classified as high to very high (0.82≤ICC≤0.97), and the SEM and MDC were not different. Another limitation was the 1-2-week interval between the two evaluations days used to determine the intra-rater reliability, even in individuals receiving rehabilitation. Although the authors did not expect changes in muscle strength in the subacute phase of stroke within this interval, the fact that some individuals were in rehabilitation programs could potentially have modified muscle strength and influenced the results of intra-rater reliability. Nevertheless, the authors emphasize that the results were satisfactory for intra-rater reliability, which suggests that this possible influence did not occur or undermine the results. In addition, to ensure the internal validity of the study, a recorder was included to perform the reading and recording of values, which is not common in clinical practice. Finally, the sample loss on the second day of evaluation was greater than expected, and only 24 individuals were evaluated on the second day. However, this sample size is higher than the minimum sample size value (n=14). In addition, the values of the intra-rater reliability for all the measures were classified as high to very high (0.74≤ICC≤0.99), except for the first trial of the non-paretic pulp-to-pulp pinch that was classified as moderate (ICC=0.64), which is also considered as adequate.

The portable dynamometers presented adequate reliability in the subacute phase of stroke, and therefore, can be used in clinical practice to measure muscle strength in these individuals if the individuals are able to do the tests. In addition, only one trial measurement of handgrip, pinch, and trunk strength is adequate. The use of only one trial has the advantage of requiring a shorter evaluation period, reducing possible effects of fatigue, loss of attention, and discomfort. In this specific population, the possibility of using only one trial after familiarization is even more important due to the characteristics of the muscle fatigue or an inability to do the test commonly observed^32^. Moreover, the MDC presented in this study could be used to decide whether a change in muscle strength is real.

## Conclusion

The portable dynamometers presented adequate inter-rater and intra-rater reliability for the measurement of handgrip, pinch grip (i.e. pulp-to-pulp, tripod, and lateral pinch), and trunk muscle strength in individuals in the subacute phase of stroke, with the use of only one trial after familiarization. The values associated with the SEM and MDC were low and should be used for drawing conclusions when evaluating muscle strength in this population.
